# Supplementary material for: Brewer’s Spent Yeast Cell Wall Polysaccharides as Vegan and Clean Label Additives for Mayonnaise Formulation
Source: Molecules. 2023 Apr 17;28(8):3540. doi: 10.3390/molecules28083540 (PMC10146781; doi:10.3390/molecules28083540)
Supplement: Supplementary file 1 [file molecules-28-03540-s001.zip › molecules-2311196-supplementary.pdf]

Brewer's Spent Yeast Cell Wall Polysaccharides as Vegan and Clean Label Additives for Mayonnaise Formulation

Sofia F. Reis [1], Pedro A. R. Fernandes [1,2], Vítor J. Martins [1], Sara Gonçalves [1], Luís P. Ferreira [2], Vítor M. Gaspar [2], Diogo Figueira [3], Diogo Castelo-Branco [3], João F. Mano [2], Manuel A. Coimbra [1] and Elisabete Coelho [1,*]

Table S1. Texture parameters of mayonnaise models with different additives including BSY extracts.

Additive	Firmness (g)	Consistency (g.s)	Cohesiveness (g)	Work of Cohesion (g.s)
Xanthan gum	35±1	875±83	-34±1	-48±1
Freeze-dried egg yolk	8.0±0.1	229±10	-6.0±0.1	-1.5±0.1
Modified starch	8.3±0.7	176±14	-6.4±0.2	-1.5±0.1
Sn 1M	8.7±0.2	214±28	-6.7±0.2	-1.7±0.1
Sn 4M	7.9±0.0	193±15	-6.0±0.1	-1.4±0.1
pp 4M	8.4±0.2	209±15	-6.6±0.2	-1.7±0.1
pp 200°C	8.1±0.1	210±13	-6.4±0.1	-1.6±0.1

Table S2. Texture parameters of a standard mayonnaise and mayonnaises with addition of BSY extracts.

Additive	Firmness (g)	Consistency (g.s)	Cohesiveness (g)	Work of Cohesion (g.s)
Standard	96±3	2709±167	-114±4	-169±6
Sn 1M	14±1	219±30	-16±2	-7.7±1.6
Sn 4M	11±0	171±2	-10±0	-2.4±0.1
pp 4M	63±1	1723±89	-63±2	-87±5
pp 200°C	12±0	188±2	-11±0	-2.6±0.2
